# Environmental Justice in Greater Los Angeles: Impacts of Spatial and Ethnic Factors on Residents’ Socioeconomic and Health Status

**DOI:** 10.3390/ijerph19095311

**Published:** 2022-04-27

**Authors:** Yuliang Jiang, Yufeng Yang

**Affiliations:** 1 Landscape Justice Initiative, School of Architecture, University of Southern California, Los Angeles, CA 90089, USA; yuliangj@usc.edu; 2 Stillwater Sciences, Los Angeles, CA 90013, USA; 3 Space Syntax Laboratory, The Bartlett School of Architecture, University College London, London WC1H 0AY, UK

**Keywords:** distance to green space, spatial centrality, pollution distribution, racial equity, structural equation modeling, space syntax

## Abstract

Environmental justice advocates that all people are protected from disproportionate impacts of environmental hazards. Despite this ideal aspiration, social and environmental inequalities exist throughout greater Los Angeles. Previous research has identified and mapped pollutant levels, demographic information, and the population’s socioeconomic status and health issues. Nevertheless, the complex interrelationships between these factors remain unclear. To close this knowledge gap, we first measured the spatial centrality using sDNA software. These data were then integrated with other socioeconomic and health data collected from CalEnvironScreen, with census tract as the unit of analysis. Finally, structural equation modeling (SEM) was executed to explore direct, indirect, and total effects among variables. The results show that the White population tends to reside in the more segregated areas and lives closer to green space, contributing to higher housing stability, financial security, and more education attainment. In contrast, people of color, especially Latinx, experience the opposite of the environmental benefits. Spatial centrality exhibits a significant indirect effect on environmental justice by influencing ethnicity composition and pollution levels. Moreover, green space accessibility significantly influences environmental justice via pollution. These findings can assist decision-makers to create a more inclusive society and curtail social segregation for all individuals.

## 1. Introduction

As stated in the Rio de Janeiro Declaration, a healthy environment is a fundamental right of all Earth’s inhabitants [1]. To ensure this right, grassroots activists and authorities are running a burgeoning worldwide social movement for environmental justice [2]. Environmental justice (hereafter, EJ) can be defined as “the fair treatment and meaningful involvement of all people regardless of race, color, national origin, or income, concerning the development, implementation, and enforcement of environmental laws, regulations, and policies” [3]. Implementing environmental justice measures is a vital component of creating and protecting a clean and healthy environment, particularly for individuals who live, work, and play close to pollutants, so that they can maintain physical and mental health [4,5]. EJ investigations enable and facilitate individuals’ voices to be heard by the decision-makers and thus promote everyone thriving in a healthy environment [6].

Our research focuses on EJ in greater Los Angeles. Abundant studies have documented environmental injustices in this region, such as the historical evolution of discriminatory pollution patterns, e.g., [7,8,9], and the racial inequality in access to urban green space (UGS), e.g., [10,11]. Furthermore, centrality is also related with EJ. Derived from graph theory, centrality was first developed in social network analysis for measuring the importance of a node in a network [12]. The spatial centrality can be decoded by space syntax for measuring the configuration of buildings and settlements mathematically: the higher the centrality value, the more integrated and accessible the street; the lower the value, the more segregated and inaccessible the street [13]. These mathematical measures often include some major indicators such as betweenness and closeness centrality to enable a detailed representation and analysis of the city’s structure [14]. Disparities in spatial centrality have been identified to associate with accessibility of urban amenities, job opportunities, and transportation [15,16,17]; therefore, we aim to explore how spatial centrality might correlate with EJ. Through the lens of spatial attributes, previous studies have also identified socioeconomic inequities with spatial indexes including the local indicators of spatial autocorrelation (LISA) and the urban centrality index (UCI), e.g., [15,18], or geographical clustering indicators [19] in Los Angeles. In addition, existing research on poverty among racial groups [20] and health issues in ethnic groups [21] discusses prominent topics in EJ since they are pertinent to each individual’s quality of life.

Despite their meaningful findings, the previous studies have mainly focused on some specific aspects of EJ using conventional spatial variables (e.g., distance to green space and pollution burdens). However, less research has comprehensively evaluated the influences of the built environment (especially its configurational attributes, such as spatial centrality) and demographic factors on EJ outcomes in greater Los Angeles. Though research on EJ and related topics can be dated back to half a century ago, e.g., [22,23], environmental injustice problems still exist today in our society, from disproportionate pollution burdens to unequal green space accessibility. Our study not only quantitively evaluated built environment and ethnic variables and their correlation with EJ, but also qualitatively analyzed the reason behind such results, integrating with cultural and legal backgrounds in Southern California. Identifying the correlations among these topics and in-depth analyzing of them can help us and decision-makers understand how they influence EJ problems and address them accordingly.

To address the knowledge gap regarding the interrelationship between built environment, demography, and EJ, we try to answer the following research questions:

Q1: Does the built environment influence EJ?
  Q1.1: Do spatial centrality and distance to green space directly influence EJ?
  Q1.2: Do spatial centrality and distance to green space indirectly influence EJ through the mediator of pollution distribution?
Q2: Are there any ethnicities particularly (dis)advantaged in EJ in greater Los Angeles?
Q3: Does the built environment influence EJ by shaping ethnicity distribution?

By answering these questions, we aim to advance the EJ literature by examining the impact of spatial configuration, distance to green space (hereafter, DGS), pollution, and ethnic composition on the population’s socioeconomic status (hereafter, SES) and health issues in the region. We first established a conceptual framework based on literature review. We then collected and integrated data on the built environment (i.e., spatial configuration, DGS, pollution), ethnic composition, and environmental justice (i.e., SES and health issues) using census tracts as the unit of analysis. Subsequently, we constructed a structural model based on the conceptual model, followed by reliability and validity tests. Finally, we assessed both direct and indirect effects of the built environment and ethnicity on EJ outcomes through structural equation modeling (SEM). As for ethnic disparities in EJ, we collected data of all ethnic groups in our study area, including “White”, “Black or African American”, “Asian”, “Hispanic or Latino” (hereafter, Latinx), “Native American”, “Native Hawaiian and Other Pacific Islander”, and “Two or More Races” (multi-racial).

Our results indicated that although spatial centrality had minimal direct effects on EJ outcomes, it demonstrated significant indirect effects by determining the pollution levels and ethnic composition. Specifically, Latinx tend to live in more integrated areas and are more exposed to concentrated pollution, while White populations reside in more segregated areas with fewer contaminants. Furthermore, DGS illustrated small yet statistically significant direct effects on EJ. Additionally, DGS demonstrated significant indirect effects by determining the area’s pollution levels, thus further influencing residents’ health. Our results indicated that Latinx are at a greater disadvantage than other ethnicities regarding DGS. The discoveries also pinpointed the most pressing issues, such as pollution remediation for transportation corridors and environmental racism, that need to be addressed for the disadvantaged communities. These findings can help the government and planning agencies to understand and address these inequalities.

## 2. Literature Review and Conceptual Framework

In this section, we first review relevant literature on EJ, including its definition, components, indicators, and how it influences populations in the USA. Building upon this review, we then propose a conceptual framework with research hypotheses regarding the influences of spatial (e.g., spatial centrality, pollution, DGS) and ethnic variables on EJ outcomes (e.g., SES and health issues).

### 2.1. Environmental Justice

#### 2.1.1. Definition, Components, and Indicators

EJ is both a means and an objective to ensure that all people, regardless of race, national origin, or income, are protected from disproportionate impacts of environmental hazards [3]. Hence, EJ as a broad concept attempts to correct environmental racism and environmental classism. Environmental racism means racial discrimination in environmental policymaking, regulation, and law enforcement, including targeting communities of color for locations of hazardous waste disposal and harmful industry [24,25,26]. Environmental classism is the outcome and process by which environmental policy implementation has planned or unforeseen repercussions that disproportionately affect lower-income individuals, groups, or communities.

Two concepts similar to EJ need to be clarified: environmental equity and environmental equality. While environmental equity emphasizes that everyone gets the support they need, environmental equality advocates offering everyone the same support with equal treatment [27,28,29]. Compared with EJ, neither environmental equity nor environmental equality addressed the causes for the unequal treatment [30]. Nowadays, most grassroots activists and the United States Environmental Protection Agency (EPA) have abandoned the term “environmental equity” in favor of “environmental justice” [31,32], because EJ is a broader and more inclusive term that better delineates the act of “redistribution of pollution” rather than solely “prevention of pollution” [33]. Therefore, despite the fine distinction among environmental justice, equity, and equality, we adopted the concept of environmental justice in this paper to examine whether environmental inequality would cause socioeconomic inequality in greater Los Angeles.

Studies of environmental justice have employed a variety of indicators over the years. In the 1970s and 1980s, environmentally disadvantaged populations were nearly exclusively determined by economic factors such as income and unemployment [22,23]. In the 1990s, social capital became an indicator for disadvantaged communities since it relates to weak and unhealthy social tires and the lack of various benefits that social networks generate [34,35]. Since the 2000s, lack of social inclusion, defined as the ability to participate in society through access to services and be heard, has become an indicator for disadvantaged populations [36]. As indicators for EJ continued to evolve, inequity in the distribution of environmental hazards is also a major concern for marginalized groups, including indigenous people and communities of color [37,38]. The evidence of health risks from industrially contaminated sites has been documented since the early 1990s [39,40]. A growing number of studies have revealed that air pollution is a severe threat to human health that can induce many diseases. Unfortunately, certain ethnic groups are the dominant victims of these environmental risks [41,42].

#### 2.1.2. Environmental Justice in the United States and Other Countries

The concept of EJ was first established in the United States in the 1980s and then spread worldwide, although similar environmental concerns had been raised before in Europe [43,44]. Globally, considerable inequities exist in terms of pollution exposure and access to environments that sustain inhabitants’ health and well-being [45]. The difference in EJ research between Europe and the US “relies on a different cultural and legal background of public policy” [46] (p. 1849), as researchers have discovered that environmental justice concerns are correlated with cultural background and the community’s awareness of protecting their own rights [43]. EJ studies in Europe are primarily focused on mapping of contaminants and their health impacts on different socioeconomic classes [46]. Communities living in or near contaminated areas are characterized by a high percentage of ethnic minorities and a low SES, resulting in growing EJ challenges [47,48]. For instance, a study in Netherlands focused on distributive justice has revealed that presence and quality of green space differ by neighborhoods’ SES [49]. Another study in Finland has pivoted on how action research can facilitate EJ via social learning, land-use planning, and legislation [50]. Other studies have exemplified similar social-spatial disparities in the UK [51], Germany [52], France [53], and the Czech Republic [54].

In contrast, the evolution of EJ in the United States was built upon a series of social movements, and the topic was officially established as a primary objective of the US government through an Executive Presidential Order in 1994 [55,56,57]. Racial segregation is the deliberate separation of individuals in everyday life into racial or other ethnic groups. It was prevalent in many cities around the country in the early twentieth century, such as issues of unequal housing, partial resource distribution, and biased living environment [7]. There is a long history of grassroots advocacy for EJ in African American [43] and Latinx communities [58]. However, widespread evidence of racial and residential segregation still exists [59]. Although communities of color have been fighting against racism for a long time, when it comes to environmental benefits, White privilege is still manifested in better resources and more convenient amenities, particularly in Southern California [60].

Abundant investigations of EJ have focused on living standards, social rights, and social inclusion based on demographics [61]. Through studying the history and in-depth interviews, research shows that Chicanos who face different types of “structural or institutionalized inequality,”—such as racism, economic inequality, and a lack of legal status in certain circumstances—develop their social movement around an environmental concern [58]. Drawing from participant observation, interviews, and digital ethnography, Huante identified gentrification as a racial issue that encouraged new racialization perpetuating unequal growth along racial lines, with Latinx as the primary victims [62]. Furthermore, a comprehensive investigation of urban park system quality examined the most populated 100 cities in the US through “ParkScore.” It substantiated that those wealthier and Whiter cities have better park systems than less affluent and more ethnically diverse (particularly Latinx and Black) cities [63]. These studies validated that Latinx and Black populations are the ethnicities that have suffered the most from environmental injustice in the US, while in Europe, the victims are diverse [47].

In addition, various case studies in Los Angeles have revealed the uneven distribution of resources across the urban setting, indicating that marginalized communities have less access to several opportunities and resources [15,16,17,18,19]. These investigations employed a range of indicators, such as local indicators of spatial autocorrelation, an urban centrality index, or geographical clustering indicators. Though the primary research objective of these studies was not directed toward environmental justice, the results confirmed the inequity of resource distribution.

Though the previous studies have insightfully explored EJ with various angles and approaches, there are a few knowledge gaps. First, they typically measure EJ problems through one of two indicators, such as income, social inclusion, or green space accessibility. None of them, to our knowledge, have synthetically integrated multiple indices to investigate EJ holistically. Second, the potential impact of spatial centrality on EJ has been overlooked in the existing studies, though space syntax has been developed to provide spatial explanations for social issues. Our research is thus aimed to close these knowledge gaps by employing a wide range of indices (including spatial centrality) to understand EJ problems comprehensively.

### 2.2. Conceptual Framework and Hypotheses

#### 2.2.1. Distance to Green Space, Pollution Distribution, and Environmental Justice

DGS for an individual living in an urban setting influences environmental justice. Plentiful research has demonstrated the benefits of accessing nature [64,65], particularly for mental well-being [66,67,68]. For example, staying in nature can effectively reduce mental stress [69,70]. Furthermore, studies have also shown that the closer the housing is to parks, the higher its price [71,72]. This high housing price may further exacerbate people’s economic burden and worsen social segregation and environmental classism.

In addition to the direct effect, DGS also indirectly influences EJ by countering the pollution level. For example, among many other studies, Cohen et al. suggested that one of the myriad benefits of urban parks is that they can reduce noise and air pollution levels [73]. Similarly, another study demonstrated that degrading the quantity and size of green space has a detrimental impact on the air quality and microclimate nearby [74]. Exposure to air pollution, mainly particulate matter (PM), has been found to trigger asthma attacks [75] and increase the risk of cardiovascular death after a heart attack [76,77]. According to this evidence above, we propose:

**Hypothesis** **1.** *Increased distance to green space directly and positively influences EJ problems.*

**Hypothesis** **2.** *Increased distance to green space directly and positively influences pollution levels.*

**Hypothesis** **3.** *Pollution level directly and positively influences EJ problems.*

**Hypothesis** **4.** *Increased distance to green space indirectly influences EJ problems* *, mediated by pollution level.*

#### 2.2.2. Spatial Centrality, Pollution Distribution, and Environmental Justice

Cities by nature bring various people and activities together [78], while spatial segregation exacerbates existing socioeconomic inequalities [79]. To comprehend a diverse range of socio-spatial behaviors including this housing pattern, one needs to examine the mobility patterns. Transportation infrastructure illustrates the range of ways that mobility may have an impact: segmenting communities, connecting populations, and/or providing areas for interaction or conflict [80]. Research has found that places with greater centrality tend to have more opportunities, urban amenities, and open spaces [15,16,17], and, therefore, higher property value [81], which are often occupied by ethnic majorities.

Transportation-related pollution is highest along busy roads. A London-based study found that road traffic is the most significant source of air pollution, and the closer to roads, the higher pollution levels are [82]. Likewise, a Boston-based investigation found that pollution is concentrated in areas with the most traffic congestion [83]. In addition, research has identified that pollution is particularly concentrated in disadvantaged communities. Living near highways or heavily trafficked roads increases the chance of having low-birth-weight (LBW) infants [84] and worsens asthma symptoms in children [85]. Furthermore, Finkelstein et al. suggested that people with low income exposed to air pollution had higher death rates than those with higher incomes exposed to similar levels of air pollution [86].

According to the evidence above, we thus propose:

**Hypothesis** **5.** *Ethnic minorities tend to live in more segregated areas, while majorities are prone to live in more integrated areas.*

**Hypothesis** **6.** *Spatial centrality is negatively associated with EJ problems.*

**Hypothesis** **7.** *Roads with higher spatial centrality induce heavier air pollution.*

**Hypothesis** **8.** *Spatial centrality influences EJ problems via the mediator of pollution.*

#### 2.2.3. Spatial Centrality, Ethnicity, and Environmental Justice

European studies have found that ethnic minorities as homogeneous groups, often the impoverished population, are segregated in the society and they live in relatively isolated areas in the city compared with the majority [78,87]. In greater Los Angeles, case studies have revealed that marginalized populations are the primary victims of pollution. Populations of color tend to live in communities with more exposure to pollution, e.g., [7,8], with vehicle traffic accounting for half of the total [88]. This has reduced the life expectancy of affected communities by on average 12 years compared with the much more affluent surrounding communities [9]. Studies have clearly pointed out that hazardous waste has mainly been distributed towards African American, Latinx, and disadvantaged communities, while White populations live farther away from environmental pollutants [89]. Moreover, disadvantaged communities, especially neighborhoods of color, tend to have lesser amounts of, and longer travel distances to, green space [63,90]. These data demonstrate that ethnic inequalities have a significant consequence on the health of disadvantaged communities, which are often people of color [91].

Accordingly, we propose:

**Hypothesis** **9.** *Neighborhoods of color are more influenced by EJ problems.*

**Hypothesis** **10.** *Spatial centrality influences EJ problems via the mediator of ethnicity.*

#### 2.2.4. Hypothesis Summary

In summary, numerous studies have investigated the relationship among EJ factors. Thus, based on the review of existing literature on environmental justice, pollution, SES, ethnicity, greenspace and park accessibility, spatial centrality, and health issues, we proposed ten hypotheses within the research scope.

However, few pieces of literature have discussed EJ integrating all these aspects in greater Los Angeles. There is a need to analyze spatial centrality and better understand how it affects EJ, especially in the context of other influences, including environmental factors, demographics, and health. To the best of our knowledge, this study is one of the first to understand EJ in a more systematic way that includes spatial centrality and provide a comprehensive evaluation framework for future studies.

## 3. Methods

**Figure 1:**
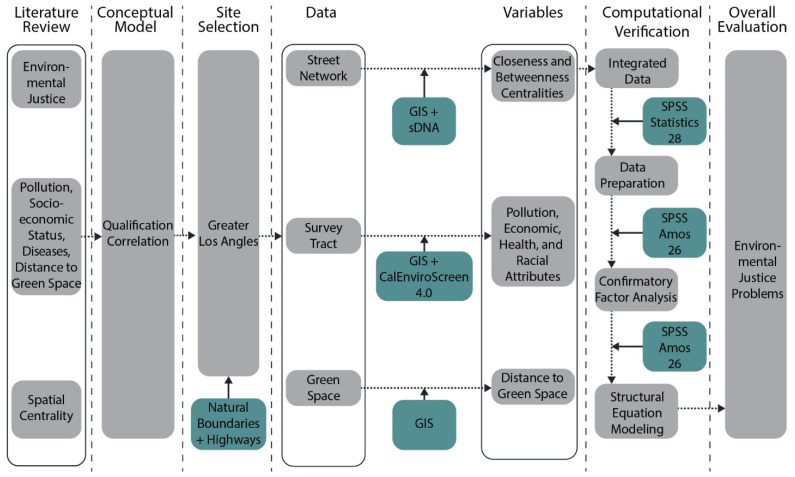
Analytic process of environmental justice evaluation in greater Los Angeles.

Figure 1 illustrates the analytic process of our research. Firstly, we defined the study site and model area. We then collected information and measured spatial centrality, DGS, other pollution, and population demographics from multiple datasets, including TIGER/Line Shapefile, United States Forest Service (USFS), and CalEnviroScreen 4.0. Subsequently, we prepared the data and conducted the reliability test. In the last step, we used structural equation modeling (SEM) to test the conceptual model.

### 3.1. Defining the Study Area

Our analysis focuses on greater Los Angles. This region has both urbanized areas and natural zones, including mountains and shorelines. It has developed highway networks and various road networks: highly accessible in downtown Los Angeles while less accessible near the mountains. In addition, green space is unevenly distributed in various locations: sparse in urbanized regions whereas aggregated near the peripheries of the metropolitan area. Los Angeles has a very diverse population, and signs denote this situation: Koreatown, Chinatown, Thai Town, Little Tokyo, Little Ethiopia, and Little Armenia are located throughout the city. Such variances on land use, road network density, and ethnic composition within the research area enable us to test how spatial centrality and DGS impact EJ and how people of different ethnicities may be affected by injustice.

**Figure 2:**
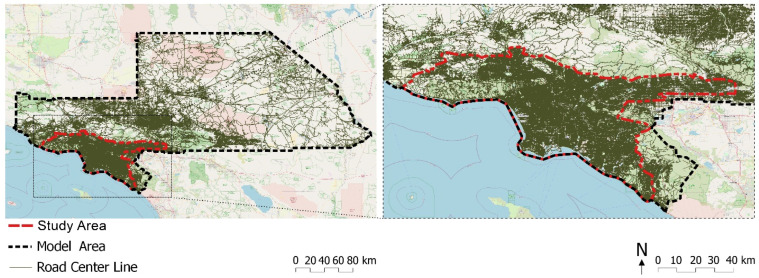
Southern California model area and greater Los Angeles study area.

Such sprawling urban settings have invited multiple investigations on spatial centrality. A proper study area is critical for modeling spatial centrality attributes. The critical point is to reduce the “edge effect”: the edge of segment models “appears disproportionally segregated to the fact that streets on the edge of the map are not connected onwards” [87] (p. 73). To avoid this effect on our outcomes, we defined our study area by natural boundaries (e.g., mountains, shorelines, and highways) and created a large model in which the study area is embedded (see Figure 2). The study area (7270 km^2^) intentionally included some state parks and mountains to investigate if these green spaces impact EJ spatially.

### 3.2. Data Collection and Measurement

This subsection introduces the data and variables used to verify our research hypotheses. Data on pollution, ethnicity, and EJ were collected from CalEnviroSceen 4.0 (Office of Environmental Health Hazard Assessment [OEHHA], Sacramento, CA, USA). This platform integrates information from federal and state sources and provides a comprehensive GIS database for each census tract in California regarding environmental concerns, health issues, and SES [21]. The CalEnviroSceen version 4.0 that we adopted combines the most recent publicly available data (2017–2021) for all indicators and refines the calculation of some indicators to more accurately represent environmental conditions or a population’s sensitivity to environmental contaminants.

#### 3.2.1. Measuring Pollution Burden

Pollution burden variables are collected from CalEnviroSceen 4.0, which incorporates two components: exposures (ozone, particulate matter [PM] 2.5, diesel PM, drinking water contaminants, lead in housing, pesticides, toxic releases from facilities, and traffic) and environmental effects (cleanups, groundwater threats, impaired waters, and solid waste). For each indicator, the measures take a mean value for a period of time to better represent a central tendency. Each indicator has a raw value and is transformed into a percentile (0–100), and then, the average component score for both exposure indicators and environmental effects indicators are individually calculated. The ultimate pollution burden is defined as the average of the scores for its two components, with the environmental effects component weighted half as much as the exposure components [22]. Thus, an area with high pollutant exposures and large environmental effects has a high pollution burden score.

#### 3.2.2. Measuring Ethnicity Composition

Demographic data were also obtained from CalEnviroSceen 4.0, which draws data from the American Community Survey (ACS) 2019 5-year estimate by the US Census Bureau. This California-based survey included about 39 million people and 14.2 million families living in California’s urban, suburban, and rural areas spread over 163,000 square miles [92]. The dataset provides the composition (in percentage) of the following seven ethnic groups within each census tract: “White”, “Black or African American”, “Asian”, “Hispanic or Latino” (Latinx), “Native American”, “Native Hawaiian and Other Pacific Islander”, and “Two or More Races” (multi-racial).

#### 3.2.3. Measuring Environmental Justice Problems

We characterized environmental justice problems based on inhabitants’ socioeconomic and health status. SES was measured using data on educational attainment, housing-burdened low-income households, and poverty collected from CalEnviroSceen 4.0. These three measurements are significant indicators of SES: limited educational attainment can lead to economic hardship, stress, fewer occupational opportunities, a lack of social support, and decreased access to health-protective services [21]. Annually, the US Census Bureau collects data on educational attainment and poverty through the American Community Survey (ACS). Census tracts were sorted by percentage of the population over age 25 with less than a high school education (5-year estimate, 2014–2018) and percent living below two times the federal poverty level (5-year estimate, 2014–2018). In terms of housing burdens, data from the Housing and Urban Development (HUD) Comprehensive Housing Affordability Strategy (CHAS) were used to identify areas where high housing expenses may strain low-income households. OEHHA tracks families earning less than 80% of the HUD area median family income and spending more than 50% of their income on housing expenditures by county. The indicator considers the geographical cost of living for homeowners and renters. Census tracts that met the criteria were sorted and given percentiles based on where they were in the distribution.

Regarding health status, we captured data from CalEnviroSceen 4.0 on asthma (rate per 10,000 people), cardiovascular disease (heart attacks per 10,000 people), and low-birth-weight (LBW) infants (percentage). Because pollutants may both cause and aggravate asthma, the prevalence of the disease is efficient to measure the general population’s susceptibility to environmental stresses [75]. In addition, those who already have cardiac disease or have had a heart attack react differently to pollution’s effects than people who do not. In addition, exposure to high air pollution increases mortality after a heart attack [76,93]. Furthermore, specific environmental and social stressors enhance the risk of LBW. The probability of having a LBW term infant is higher if pregnant women live near a highway or heavily used road [84]. Therefore, it may be used as a barometer to measure the cumulative impact of environmental and social stresses since the environmental burdens are concentrated on populations of color.

Both asthma and cardiovascular disease data are from “Emergency Department and Patient Discharge Datasets from the State of California, Office of Statewide Health Planning and Development (OSHPD)” [21] (pp. 144, 149). Tracking California conducted a series of computations to estimate the rate per 10,000 people based on records for ED visits occurring during 2015–2017 for patients listed as residing in California and having a principal diagnostic of asthma and heart attack. Ultimately, the spatially modeled apportionment rate was used to rank the census tracts and assign percentiles depending on their position in the distribution of the total population [21]. In comparison, low-birth-weight data are from the California Department of Public Health (CDPH) [21]. Low birth weight was computed using California birth records as the percentage of live, singleton newborns weighing less than 2.5 kg in 2009–2015. After a series of geographic location analysis and data computations, similarly, census tracts were sorted and assigned percentiles.

#### 3.2.4. Measuring Spatial Centrality

We measured the closeness and betweenness centrality of street networks using sDNA [94], a spatial network analysis toolbox for GIS and Python. The closeness centrality represents the accessibility from a road to the rest of the roads within a given radius [95]. High closeness centrality indicates a high likelihood of people coming to the place, namely, the to-movement potential [96,97]. Analogously to the approach utilized by Sun et al. [98], we used closeness to measure the transport accessibility: “from every space to every other space within the network where the cost is calculated as a function based on the configuration or geometry of the grid” [99] (p. 8021:2). The formula is as follows:

(1) $$\mathrm{Closeness}\left(i\right)=\frac{1}{l_{i}}=\frac{n}{\sum_{j}d_{ij}},$$

where the length of the shortest path from i to j is denoted by $d_{ij}$. The mean distance from i to j over all nodes j in the network is $l_{i}$.

Betweenness centrality depicts the possible through-movement of any road link that pedestrians or automobiles might choose from [100]. High betweenness centrality indicates a high likelihood of people moving through a place on the way from one location to another [98]. It is often measured as the diversion ratio of the shortest routes between neighboring nodes that traverse through the node of interest [100,101,102,103]. Similar to Zhang et al.’s method [96], we measured betweenness centrality using the equation below:

(2) $$\mathrm{Betweenness}\left(x\right)=\sum_{yz}n_{yz}^{x},$$

where $n_{yz}^{x}$ is one if *x* lies on the shortest path from *y* to *z* and zero if it does not.

We extracted the road centerline from TIGER/Line Shapefile (metadata updated in November 2020) and employed it as the base street network database. A series of radii were used for computing the closeness and betweenness centrality, ranging from local (400 m, 800 m) and city (2000 m, 5000 m) to regional scale (25,000 m).

#### 3.2.5. Measuring Distance to Green Space

**Figure 3:**
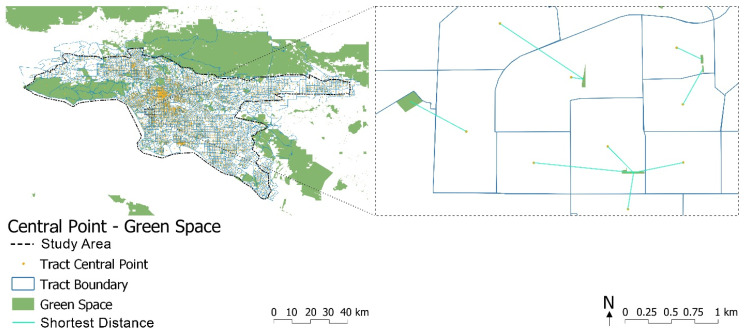
Central point of each census tract and the compiled green space. Note that the small green patches are all community gardens in this specifically enlarged area.

GIS data on park and green areas within the study area were collected from the United States Forest Service (USFS) and the County of Los Angeles Department of Parks & Recreation, which integrates all local parks, regional recreation parks, regional open space, and natural areas (see Figure 3). Similar to Zhang et al.’s method [104], we extracted the centroid of each of the 3041 survey tracts in the study area and calculated the distance from the centroid of each survey tract to that of the nearest green space in the Quantum geographic information system (QGIS) using the command “distance to the nearest hub”, as shown in Figure 3.

### 3.3. Analysis Approach

**Figure 4:**
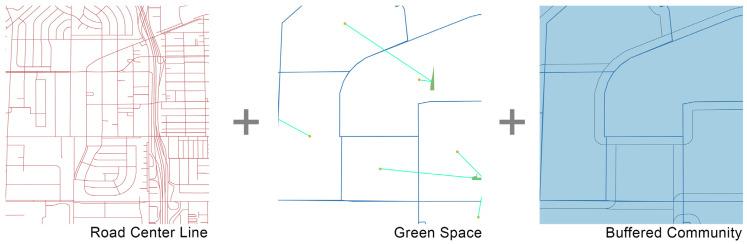
Integration of three datasets.

The next step was to integrate various datasets using the census tract as the unit of analysis (see Figure 4). A shapefile was downloaded from CalEnvironSceen 4.0 containing the environmental burdens, economic, health, and demographic attributes for all 3041 survey tracts in our study area. To integrate the street centrality attributes, we created a 400-m buffer around each survey tract to include the roads and highways on the edge of the tract. Subsequently, we took the average centrality value of all street segments within the buffer area using the “join attribute by location” command in QGIS.

Following the data integration was the statistical analysis. We first conducted the multicollinearity test in SPSS Statistics 28. Multicollinearity issues were determined between closeness and betweenness centrality at five different radii, with variance inflation factors (VIF) above 10. Consequently, we only retained the centrality variables measured at a 25,000 m radius for further SEM analysis because they demonstrated the strongest correlation with SES and health issues and showed the lowest VIF (4.682).

Similarly, ethnicity compositions in demographic data also demonstrated a multicollinearity issue. Initially, we concluded all seven ethnicities (Latinx, White, African American, Native American, Asian, Pacific Islander, and Two or More Races). Their VIF ranged from 3.977 to 13.597, suggesting the existence of the multicollinearity issue. As a result, we only kept three ethnicity types (Latinx, White, and Two or More races) with VIF values lower than 5.

Furthermore, since SEM assumes that the variables follow a normal distribution, data cleaning and transformation are needed [105]. We first excluded all outliers in each attribute, yielding 2826 remaining samples. We also performed logarithmic transformation for the closeness centrality, betweenness centrality, and distance to green space data before SEM to meet the normality requirement. All other data adopted from CalEnviroScreen 4.0 were normally distributed and ready to be calculated in the SEM (summarized in Table 1).

**Figure 5:**
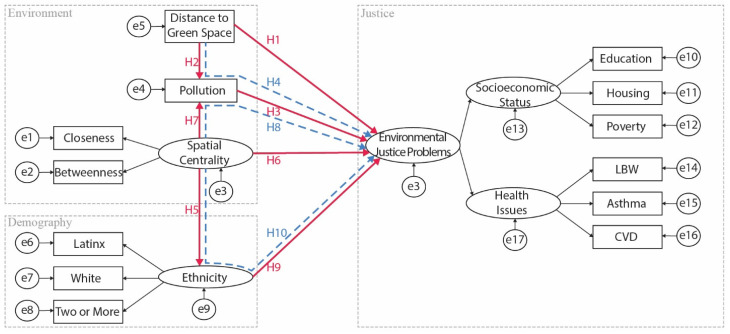
Structural equation model for measuring environmental justice. LBW stands for low-birth-weight infant and CVD represents cardiovascular disease; the solid red line represents direct effect; the blue dash line indicates indirect effect; H1 to H10 stand for corresponding hypotheses; e stands for the measurement error.

Subsequently, the cleaned and transformed dataset was entered in SPSS AMOS 26 to construct a structural model (Figure 5). Given that this research measures the latent variable ‘EJ problems’ through two other latent variables (namely, inhabitants’ socioeconomic and health status), we followed recommendations by Anderson and Gerbing [106] using a two-step approach. The first step is to analyze the measurement model (first-order measurement—SES and health issues in our case), and the second step is to analyze the path model (second-order measurements—the paths between ethnicity to EJ problems and spatial centrality to EJ problems). Confirmatory factor analysis (CFA) was performed in the first-order measurement to examine the structural validity and the convergent validity of this model [107]. A series of indices were used, including χ^2^/df, RMSEA, P, SRMR, NFI, and CFI. Additionally, the average variance extracted (AVE), r^2^, and composite reliability (CR) were used for verifying the convergent validity. Having tested the validity and reliability, in the second-order measurements, we computed the structural model, basing it on the measurement model found in the first-order measurement. Next, further structural equation modeling (SEM) analysis was computed with the maximum likelihood estimation. SEM is a powerful analytical tool, which enables the modeler to simultaneously assess a range of regression equations [108]. SEM also enables a third variable, known as a mediator, to indirectly alter the relationship between two constructs. Consequently, the impact of the two constructions will be intervened by the presence of this third variable (pollution and ethnicity in our case) [109].

Lastly, we used a series of diagrams obtained from our GIS analysis to evaluate each variable in the built environment, demography, and EJ and how they respond to the hypothesis. Most of these variables are single data that can be directly symbolized and presented via GIS, whereas the EJ data is a compound variable; thus, we calculated the mean of its six indicators (education, housing, poverty, LBW, asthma, and CVD), and used GIS to display the data. Diagrams were then analyzed and evaluated with some previous studies in the organized categories.

## 4. Results

This section first reports the reliability and validity test results of the CFA model and the results of SEM. Direct and indirect effects between variables are then evaluated to test our hypotheses.

### 4.1. Reliability and Validity Testing

#### 4.1.1. First-Order Measurement Model Evaluation

**Figure 6:**
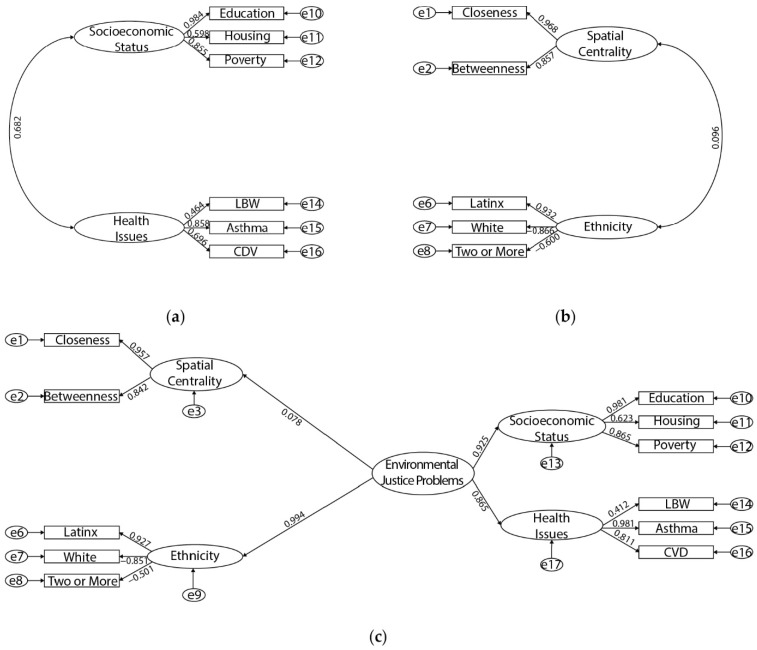
Stepwise CFA: (**a**) first-order measurement model; (**b**) second-order influential variables model; (**c**) second-order measurement model.

Since our model includes a second-order latent variable—EJ—, a second-order CFA model was used to test the assumption that the correlations among a set of first-order factors are accounted for in one or more higher-order factors [110]. Thus, the CFA was computed in two steps for the first- and second-order measurements, respectively. The first-order measurement of our model (Figure 6a) presents a sufficient goodness-of-fit to the data, CFI = 0.996, NFI = 0.996, and SRMR = 0.023, based on criteria in Table 2, and the correlation between SES and health issues is strong (*β* = 0.682).

After the first-order model fit evaluation, we then utilized this model to examine the convergent validity for the first-order constructs (see Table 3). The convergent validity was evaluated through the factor loadings, r^2^, AVE, and CR, for measurement models as in Table 3, with the threshold for the good fit at 0.5 [111], 0.26 [112], 0.5 [113], and 0.65 [114], respectively. Despite the AVE for the health issues being slightly below the benchmark of 0.05, all other indices suggested a sufficient fit.

Brown suggested that a discriminant validity test for the first-order latent variables is unnecessary since the two first-order latent variables (SES and health issues) are categorized under the second-order measurement, EJ [110]. In addition, there is no need to run a discriminant validity test between the second-order latent variable and the first-order measurements [110].

#### 4.1.2. Second-Order Measurement Model Evaluation

Table 2 shows the goodness-of-fit of the second-order measurement model, suggesting that the model exhibits an acceptable fit to most indices, although the *p*-value does not reach the acceptable baseline. Good model fit expects this value to be above 0.05 [115]. However, the *p*-value tends to be significant for models with large sample sizes [116], as in this case (sample size = 2826). Therefore, we conclude that the second-order measurement model showed an adequate fit.

**Table 2:** Indices of the first- and second-order measurement model fit.

	χ^2^/df	RMSEA	*p*-Value	SRMR	NFI	CFI ^1^
Criteria	2.0–5.0 [107]	<0.08 [115]	>0.05 [115]	<0.09 [107]	>0.9 [117]	>0.9 [118]
First-Order Model	2.148	0.069	0.000	0.023	0.996	0.996
Second-Order Model	2.399	0.077	0.000	0.062	0.917	0.928

^1^ RMSEA, root mean square error of approximation; SRMR, standardized root mean square residual; NFI, Bentler-Bonett normed fit index; CFI, comparative fit index.

We evaluated the convergent validity of EJ’s influential variables (spatial centrality and ethnicity) as in Figure 6b and second-order measurement model as in Figure 6c. Only one factor loading (low birth weight, 0.464) is less than the 0.5 threshold but very close, demonstrating an adequate convergent validity [107,111]. In addition, almost all average variance extracted (AVE) exceeded 0.5 [113], except health issues being very close (0.021 shortage). All composite reliabilities exceed the 0.65 benchmarks [114]. The AVE and CR suggest that the model has an adequate convergent validity. Furthermore, after completing the first- and second-order measurement model convergent validity tests, we calculated and found that the SEM showed a sufficient goodness-of-fit to the data: CFI = 0.940, NFI = 0.938, SRMR = 0.079.

**Table 3:** First- and second-order measurement model convergent validity.

Variable Path			Factor Loadings	Squared Multiple Correlations (r^2^)	Average Variance Extracted (AVE)	Composite Reliability (CR)
**First-Order Measurement**						
Socioeconomic Status	→	Education	0.984	0.968	0.686	0.863
Socioeconomic Status	→	Housing	0.598	0.358		
Socioeconomic Status	→	Poverty	0.855	0.731		
Health Issues	→	Low-Birth-Weight Infant	0.464	0.215	0.479	0.723
Health Issues	→	Asthma	0.858	0.736		
Health Issues	→	Cardiovascular Disease	0.696	0.484		
**Second-Order Measurement**						
Environmental Justice Problems	→	Socioeconomic Status	0.962	0.925	0.837	0.911
Environmental Justice Problems	→	Health Issue	0.865	0.748		
Spatial Centrality	→	Closeness	0.968	0.937	0.836	0.910
Spatial Centrality	→	Betweenness	0.857	0.734		
Ethnicity	→	Latinx	0.932	0.869	0.659	0.849
Ethnicity	→	White	−0.866	0.750		
Ethnicity	→	Two or More Races	−0.600	0.360		

### 4.2. Test of Hypotheses

**Figure 7:**
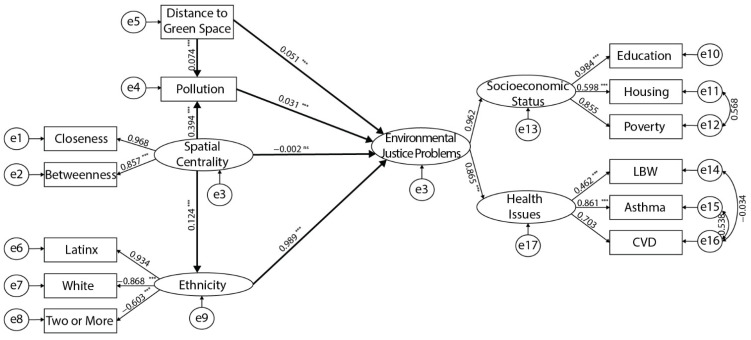
Standard coefficients of the SEM. Note that *** represents significance at 0.001 level; ns represents no significance; e stands for the measurement error.

Having passed the reliability and validity tests, SEM was validly conducted to test our hypotheses (Figure 7). Table 4 illustrates each regression path’s standard error, standardized estimate, critical ratio, and significance levels. Overall, most of the regression weights for the variables are significant.

In terms of the standardized estimate, most of the pathways showed low correlations. Among all predictors, the highest coefficient occurs in the path from ethnicity to environmental justice, reaching 0.989, which illustrated that ethnicity is a major concern for environmental justice in all hypotheses. What also stands out in Table 4 is the weak and non-significant influence of spatial centrality on environmental justice. Furthermore, pollution and DGS also exhibit weak yet significant correlations with EJ.

Our SEM diagram (Figure 7) shows that ethnicity and pollution are two mediators. Mediation can only be established in an SEM context if the total and indirect effects are significant [119]. Therefore, we further examined the total effect of spatial centrality on ethnicity, and the total effect of DGS on EJ. In addition, we assessed the indirect effect of spatial centrality via the mediator of pollution and ethnicity and the indirect effect of DGS via the mediator of pollution. These mediators generated a statistical significance in their pathways for total effect and indirect effect (Table 5), confirming that our mediation paths are valid in the SEM framework. Although the direct effect of spatial centrality on EJ is neglectable (*β* = −0.002, *p*-value = 0.825), the two mediators—pollution and ethnicity—are substantially contributing to the total effect. Notably, ethnicity (*β* = 0.121, *p*-value < 0.001) is a significantly more potent mediator of this relationship than pollution for EJ (*β* = 0.014, *p*-value = 0.007). However, in terms of correlation between DGS and EJ, the indirect effect via pollution (*β* = 0.002, *p*-value = 0.002) is trivial, making the DGS’s direct effect on EJ the primary path to comprise the total effect (*β* = 0.053, *p*-value < 0.001).

## 5. Discussion

Our findings suggest that except for ethnicity, other variables including pollution, DGS, and spatial centrality all exhibit weak effects on EJ. In terms of indirect effect, pollution is a statistically significant mediator on two pathways: between spatial centrality and EJ (H8) and between DGS and EJ (H4). Because people of color in the US tend to live in more spatially centralized areas with high pollution levels, the interactions of ethnicity and pollution lead to a higher likelihood of ethnic minorities suffering from EJ (i.e., health issues and SES). In this section, we will discuss some consistencies and disparities that appeared between our SEM results and previous research findings in more depth.

### 5.1. Direct Effect of Ethnicity on Environmental Justice (Hypothesis 9)

**Figure 8:**
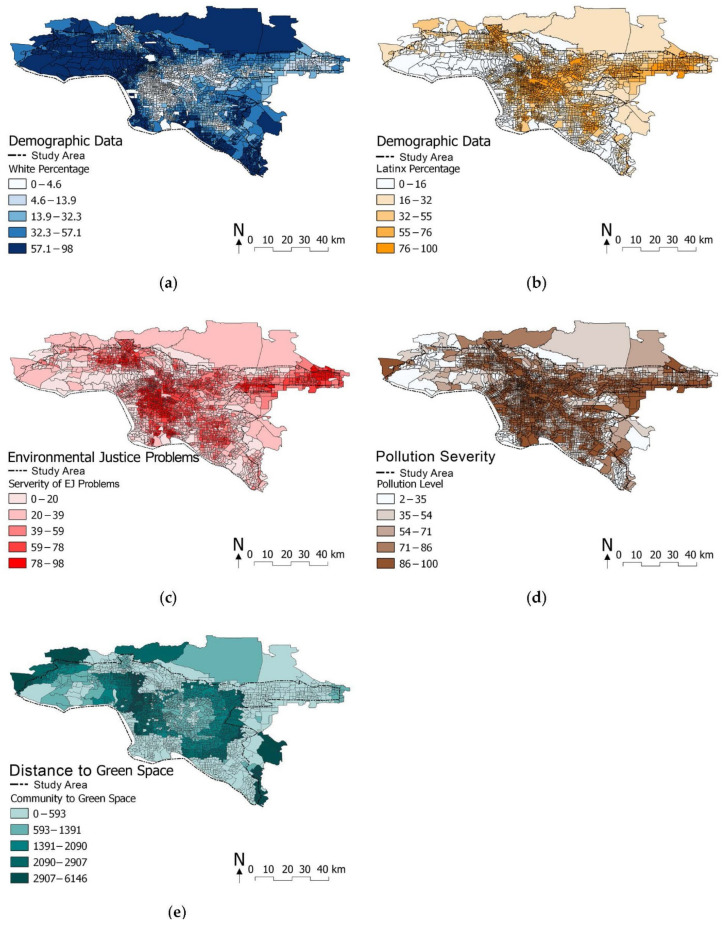
Multiple variants comparison. (**a**) White distribution; (**b**) Latinx distribution; (**c**) environmental justice problems: spatial mapping of the mean of three SES indicators and three health issue indicators; (**d**) pollution level: the darker the color, the more pollution; (**e**) distance to green space: the darker the color, the further the distance.

Our evaluation showed that ethnicity has a strong correlation with environmental justice problems (H9). This finding is consistent with abundant existing studies, e.g., [7,8,9], revealing that some ethnic minorities are at higher risk of environmental injustice such as SES and health issues, while the White population are at less risk. One can identify severe racial segregation through visual inspection (see Figure 8a–c): most White populations live in areas with less environmental injustice, while Latinx are inequitably impacted by environmental burdens.

Our finding is in line with many other studies that have also found that some ethnicities suffer from health issues caused by environmental burdens, and disparities in health for people of color have existed for a long time, e.g., [120,121,122,123]. For disadvantaged populations and people of color in California, the dangers of exposure to environmental hazards are severe: they are 61% more likely to live in an area shrouded in unhealthy air than are White populations and three times more likely to live in a county with failing air-quality grades [21,37,38]. The situation is also exemplified in Los Angeles. Based on data from the Los Angeles County Public Health Department [124], asthma-related emergency room visits by Latinxs are more than twice as high as those by Whites, and Black children have the highest rates of asthma (25%) compared with all other ethnicities. To close the health equality gap, the relationship between racism and poor health must be acknowledged [120].

### 5.2. Direct Effect of Pollution on Environmental Justice (Hypothesis 3)

We found a positive, weak yet significant correlation between pollution and environmental justice problems (*β* = 0.031), while most previous studies, e.g., [125,126,127] have suggested the uneven distribution of pollution strongly impairs environmental justice with a statistical significance. Despite the low factor loading, the correspondence between environmental justice and pollution level can be visually identified from Figure 8c,d. However, one possible reason for the weak correlation may be the fluidity of the contaminants. The pollution dataset covers liquids (e.g., drinking water contaminants) and extremely light contaminants (e.g., ozone, PM 2.5, and diesel PM) with high mobility [128]. They move around via gravity and wind, so topology and wind tunnels could influence the data accuracy. As identified by Croxford et al. [129], pollutant concentrations are highly dependent on local wind speed.

### 5.3. Direct Effect of Distance to Green Space on Pollution and Environmental Justice (Hypotheses 1 and 2)

Unlike previous studies, e.g., [73,74], that found a strong positive correlation between DGS and pollution, our study showcased a positive yet weak correlation (H2). Furthermore, the existing studies, e.g., [64,65,67,68], found a robust positive correlation between DGS and environmental injustice, whereas our study demonstrated a weak positive correlation between these two variables (H1). One possible reason for these unexpected findings could be that we measured DGS as the straight-line distance between the centroid of each tract and its nearest green space on the map. In other words, we did not precisely measure the actual walking distance between each tract and its nearest green space, but this method has been widely applied in many previous studies, e.g., [130,131].

Although the measurement does not precisely represent the actual walking distance, we can still distinguish a clear uneven distribution of DGS in the research area, with DGS (Figure 8e) corresponding somewhat with pollution level (Figure 8d), despite a weak correlation from the SEM estimation. Our result is spatially consistent with some existing DGS studies in downtown Los Angeles where there is heavier traffic, more pollution, and less green spaces in areas dominated by people of color, while in White-dominant, wealthy communities, the situation is the opposite [63,132,133]. Having access to green space means opportunities to enjoy ecosystem services, which are the varied benefits to humans provided by parks and green spaces, e.g., [134,135]. The extensive range of distance to the nearest green space means highly uneven access to ecosystem services [136].

### 5.4. Direct Effect of Spatial Centrality on Pollution, Ethnicity, and Environmental Justice (Hypotheses 7, 5, and 6)

**Figure 9:**
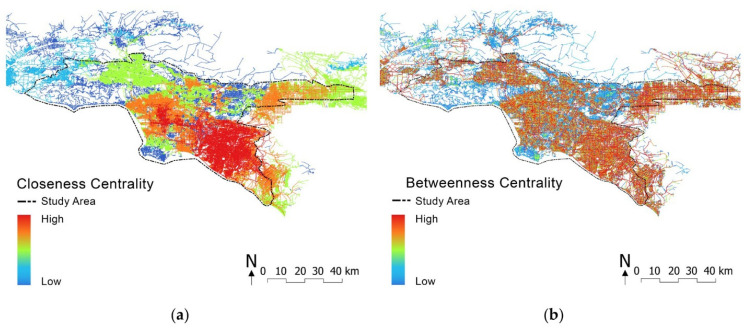
Spatial centrality of greater Los Angeles: (**a**) closeness centrality: red lines represent more central and integrated areas, whereas navy lines represent peripheral and segregated areas; (**b**) betweenness centrality: red and orange lines represent busy highways and main roads.

Our study demonstrated a significant yet surprisingly weak correlation between spatial centrality and pollution (H7: *β* = 0.394, *p*-value < 0.001). While we expected a stronger correlation because busier roads tend to induce higher pollution, there might be two possible factors that influenced our results. First, the pollution dataset was a combined dataset that also included many contaminants not from traffic, such as lead in housing, pesticides, and toxic releases from facilities. Second, traffic-induced contaminants were not only restricted to automobiles; other types of transportation such as trains and ships also released such contaminants. Consequently, the highest levels of diesel particulate matter (PM) can also be found near ports and rail yards [21] that do not have high betweenness centrality values. Croxford et al. [129] also pointed out the difficulty of measuring traffic pollution in a similar study. However, one can nonetheless notice a visual correspondence between the betweenness centrality and pollution from Figure 8d and Figure 9b, which is consistent with some previous studies [91,137].

Furthermore, spatial centrality was found weakly yet significantly correlated with ethnic composition (H5) and environmental justice (H6). We found that more ethnic minorities are clustered in the city center, where notable environmental pollution and traffic density can be witnessed (see Figure 8a,b and Figure 9a,b).

However, we discovered a pattern that is the reverse of that reported in the literature on Europe. A study has found that disadvantaged individuals in Europe—often minorities—tend to be isolated in urban space, often in ethnic enclaves [78]. In cities such as London with a developed public transit system, taking advantage of multimodal public transport to access job opportunities, vibrant social activities, and public parks in the city center increases quality of life and drives up housing costs [138,139,140,141]. Such green gentrification has accelerated displacement and resulted in only privileged groups enjoying urban amenities, demonstrating an environmental injustice.

Unlike in London where additional fees such as congestion charges and ultra-low emission zone charges are applied to disincentivize private vehicle use, a much worse pollution burden is exhibited in downtown Los Angeles. These patterns have deep roots that date back to urban planning decisions from the 20th century coupled with underlying social differences. The Regional Planning Commission established the Metropolitan Los Angeles Freeways Master Plan in 1947, and construction began in the early 1950s. The development of automobile-dominant transportation, a freeway system that could solve the region’s transportation problems and accommodate a large population, gradually abandoned unprofitable railroads such as Red Car streetcar lines [142]. Nowadays, privileged populations are able to live in places close to nature, such as Beverly Hills, Malibu, and Marina del Rey with significantly less pollution compared with the buzzing downtown Los Angeles. Considering the less-developed public transit system in greater Los Angeles, deprived populations must choose a workplace close by or spend tremendous time commuting on public transportation or in congested traffic [143]. This pattern confirmed that mobility ability is associated with inequality and exclusion [144].

### 5.5. Indirect Effect of Pollution and Ethnicity (Hypotheses 4, 8, and 10)

In addition to the direct effect, we also found the weak yet significant indirect effect of DGS on environmental justice mediated by pollution level (H4). This finding supported the theoretical study by Nowak and Heisler [145], who demonstrated that green space and parks are beneficial: park trees and plants can minimize air pollution by eliminating pollutants directly, lowering air temperatures, and reducing building energy usage in and around parks. Thus, communities closer to parks present better air quality and less energy use in air conditioning, revealing an environmental injustice for those who live far from green spaces.

Though the coefficient from spatial centrality directly to EJ is very low (H6), there is a statistically significant impact on justice through the mediators of pollution (H8) and ethnicity (H10), with ethnicity exhibiting nine times as much indirect effect as pollution on justice. These findings are consistent with considerable research that revealed that certain ethnicities are particularly vulnerable to environmental pollutants, e.g., [146]. Croxford et al.’s research [129] demonstrated that vehicular movement is strongly related to the street grid configuration, and pollution generated by traffic affects the people who live, walk, and work in the city. Places with high spatial centralities often have high traffic density, thus more pollution.

### 5.6. Limitations and Future Studies

Although this paper has provided some fruitful findings, certain limitations can be addressed in future research. First, as mentioned before, the method we utilized to assess accessibility of green space is not precise: the distance between the centroid of each tract to the nearest green space is the point-to-point straight line on the map, rather than a walking or driving distance. We did not consider the road network nor the entrance locations of the green spaces; thus, future research can use a road-based DGS measurement for a more precise assessment.

Second, constrained by CalEnviroSceen data, our unit of analysis is based on census tracts, which might induce a modifiable areal unit problem (MAUP). Future studies could adopt a finer and more appropriate analytical unit for more reliable results.

Third, aiming at a comprehensive evaluation, other influential factors are also contributing to environmental injustice in addition to the ones we evaluated—such as, community food security for life necessities [147], levels of violent and property crime [148], the amount of tree canopy cover, and urban heating [149]. More comprehensive influential factors of environmental justice can be incorporated for an exhaustive model in future studies.

Fourth, we only evaluated EJ based on SES and health issues. Other indicators, such as unemployment, linguistic isolation, and social exclusion are also worth considering in future studies to achieve a more comprehensive evaluation of EJ.

Fifth, because of the multicollinearity issue, only three types of ethnicities were included in the final evaluation. More evidence is necessary, especially for other minorities including Black or African American, Asian, Native American, Native Hawaiian, and Other Pacific Islanders that were excluded from our final evaluation. Similarly, the same issue made us keep centrality values at regional scales, while excluding local and city scales. Future studies should evaluate how multiscale centralities are associated with EJ.

Sixth, the results of this study only revealed the situation in a portion of Southern California since the study area is restricted to greater Los Angeles. Other cities in Southern California and beyond might differ due to different urban configurations, demographics, and other factors. Therefore, further investigations in other regions in the US or worldwide are needed to test the findings of this paper.

## 6. Conclusions

This study conducted an integrated investigation on environmental justice problems in greater Los Angeles. Although research on EJ and implementing EJ policies have existed for a long time, ever since the 1970s, various scholars and organizations have evidenced that EJ has not been fully achieved so far, e.g., [22,23,45,63]. A great number of studies have also confirmed that ethnic inequality exists regarding access to healthy environments, e.g., [120,124]. Returning to our research questions, the findings confirmed that ethnicity composition was closely related to EJ problems: while the White population was more advantaged, people of color (particularly the Latinx in our case) were disadvantaged, manifested in exposure to higher traffic pollution and living farther from green space (Q2). Additionally, we also found that the built environment did significantly influence EJ (Q1): although spatial centrality had a weak direct effect on EJ (Q1.1), it demonstrated a significant indirect effect by influencing ethnicity compositions (Q3) and pollution levels (Q1.2). Among the two mediators, ethnicity was the most powerful predictor for EJ. Similarly, although DGS showcased a weak yet significant correlation with EJ (Q1.1), this factor statistically significantly affected EJ via pollution (Q1.2). To summarize, the ethnic composition and pollution distribution were two statistically significant mediators between the built environment and EJ.

Our study contributes to the growing EJ literature on ethnic, economic, and well-being inequalities by holistically evaluating relationships in a theoretical framework. A noteworthy point of our study is that despite the scale of the cities, different urban developments, public transportation, infrastructure, environment, and policy can lead to opposite situations. West Central London’s parks and nice environment have attracted affluent populations to reside, while the traffic polluted Downtown Los Angeles has dispersed the wealthy society living there. Furthermore, our study has confirmed that pollution is particularly condensed along the highway, and thus, cleaner transportation methods should be considered for Los Angeles. In conclusion, the findings presented in this paper may help the government, developers, and planners work to eliminate environmental injustice in greater Los Angeles by identifying the most pressing issues and understanding the relationships of interacting factors. Ideas, protocols, and plans tackling these issues can be implemented in future legislation processes, such as increasing green space accessibility, pollution remediation, and subsidized housing programs.

## Figures and Tables

**Table 1:** Descriptive statistics for all variables.

Category	Elements	Mean	SD	Range
Environmental Attributes	Closeness Centrality ^1^	0.00047	0.00067	0.00001–0.01060
	Betweenness Centrality ^1^	1.488	4.016	0.00002–41.100
	Distance to Green Space	2947.429	1841.646	10.639–6146.646
	Pollution	68.532	22.422	1.730–100
Socioeconomic Attributes	Poverty	52.826	29.120	0–99.987
	Housing Burden	59.345	29.211	0–99.962
	Educational Attainment	54.815	30.900	0–99.962
Health Attributes	Cardiovascular Disease	51.334	26.942	0–99.227
	Asthma	48.330	27.920	0–98.779
	Low Birth Weight	51.914	29.319	0–99.949
Ethnic Attributes	Latinx	45.912	28.823	0–100 ^2^
	White	29.345	25.578	0–98.043
	Two or More Races	2.556	2.267	0–25

N = 2826. ^1^ Measured at 25,000 m radius; ^2^ 100 means there are solely Latinx residing in the surveyed community.

**Table 4:** Regression coefficients.

Hypothesis	Variable Path			Standardized Estimate	Critical Ratio	*p*-Value
H7	Spatial Centrality	→	Pollution	0.394	19.611	***
H2	Distance to green space	→	Pollution	0.074	4.289	***
H5	Spatial Centrality	→	Ethnicity	0.124	6.103	***
H6	Spatial Centrality	→	Environmental Justice Problems	−0.002	−0.222	0.852
H3	Pollution	→	Environmental Justice Problems	0.031	3.396	***
H1	Distance to green space	→	Environmental Justice Problems	0.051	5.962	***
H9	Ethnicity	→	Environmental Justice Problems	0.989	60.797	***

*** refers to significance at 0.001 level.

**Table 5:** Standardized direct, indirect, and total effect on environmental justice.

Hypothesis	Variable Path	Direct Effect (a)		Indirect Effect (b)		Total Effect	
		*β*	*p*-Value	*β*	*p*-Value	*β*	*p*-Value
H6 (a) ^1^	Spatial Centrality → Environmental Justice Problems	−0.002	0.825	0.135	0.001 ***	0.133	0.001 ***
H8 (b) ^2^	Spatial Centrality → Pollution → Environmental Justice Problems	–	–	0.014	0.007 **	–	–
H10 (b)	Spatial Centrality → Ethnicity → Environmental Justice Problems	–	–	0.121	0.001 ***	–	–
H1 (a)	Distance to Green Space → Environmental Justice Problems	0.051	0.001 ***	0.002	0.003 **	0.053	0.001 ***
H4 (b)	Distance to Green Space→ Pollution → Environmental Justice Problems	–	–	0.002	0.002 **	–	–

^1^ (a) refers to a direct effect; ^2^ (b) refers to an indirect effect; *** refers to significance at 0.001 level; ** refers to significance at 0.01 level.

## Data Availability

Not applicable.
